# Chronic Hepatitis B Genotype C Mouse Model with Persistent Covalently Closed Circular DNA

**DOI:** 10.3390/v16121890

**Published:** 2024-12-07

**Authors:** Deok-Hwa Seo, Wonhee Hur, Juhee Won, Ji-Won Han, Seung-Kew Yoon, Songmee Bae, Kyun-Hwan Kim, Pil-Soo Sung

**Affiliations:** 1 The Catholic University Liver Research Center, College of Medicine, The Catholic University of Korea, Seoul 06591, Republic of Korea; smin0904@naver.com; 2 Division of Chronic Viral Diseases, Center for Emerging Virus Research, National Institute of Health (NIH), Cheongju 28159, Republic of Korea; wonhee.her@gmail.com (W.H.); songmee@korea.kr (S.B.); 3 Department of Precision Medicine, School of Medicine, Sungkyunkwan University, Suwon 16419, Republic of Korea; 1wonjuhee@daum.net; 4 Division of Gastroenterology and Hepatology, Department of Internal Medicine, College of Medicine, Seoul St. Mary’s Hospital, The Catholic University of Korea, Seoul 06591, Republic of Korea; tmznjf@catholic.ac.kr (J.-W.H.); yoonsk@catholic.ac.kr (S.-K.Y.)

**Keywords:** Hepatitis B virus, genotype C, cccDNA, mouse model, chronic HBV infection

## Abstract

Hepatitis B virus (HBV) can cause chronic infections, significantly increasing the risk of death from cirrhosis and hepatocellular carcinoma (HCC). A key player in chronic HBV infection is covalently closed circular DNA (cccDNA), a stable episomal form of viral DNA that acts as a persistent reservoir in infected hepatocytes and drives continuous viral replication. Despite the development of several animal models, few adequately replicate cccDNA formation and maintenance, limiting our understanding of its dynamics and the evaluation of potential therapeutic interventions targeting cccDNA. In this study, we aimed to develop a mouse model to investigate cccDNA formation and maintenance. We infected C57BL/6 mice with recombinant adeno-associated virus (rAAV) carrying a 1.3-overlength HBV genome (genotype C) and collected liver tissue at various time points to assess cccDNA levels and viral replication. Our results demonstrated the successful establishment of a chronic hepatitis B mouse model using rAAV-HBV1.3, which supported persistent HBV infection with sustained cccDNA expression in hepatocytes. Serum levels of HBsAg and HBeAg were elevated for up to 12 weeks, while alanine transaminase (ALT) levels remained within the normal range, indicating limited liver damage during this period. We confirmed HBV DNA expression in hepatocytes, and importantly, cccDNA was detected using qPCR after Plasmid-Safe ATP-Dependent DNase treatment, which selectively removes non-cccDNA forms. Additionally, Southern blot analysis confirmed the presence of cccDNA isolated using the Hirt extraction method. This established model provides a valuable platform for studying the long-term maintenance of cccDNA in chronic HBV infection and offers an important tool for testing novel therapeutic strategies aimed at targeting cccDNA.

## 1. Introduction

Hepatitis B virus (HBV), a member of the *Hepadnaviridae* family, primarily infects human hepatocytes and is a major global health concern due to its role in the development of cirrhosis and hepatocellular carcinoma (HCC) [1,2,3]. Over 292 million individuals worldwide are estimated to be chronic carriers of HBV, with nearly 50% of HCC cases in the Asia–Pacific region linked to chronic HBV infection [4,5]. A functional cure for HBV is defined by the sustained loss of detectable HBsAg and the reduction in serum HBV DNA to unquantifiable levels for at least six months [6]. Current treatment options include pegylated interferon-alpha and HBV polymerase inhibitors such as nucleos(t)ide analogs (NAs), which can effectively suppress viral replication [7,8,9]. However, these therapies rarely achieve a complete cure, largely because they do not target HBV’s covalently closed circular DNA (cccDNA) [10]. cccDNA is a stable, episomal form of the HBV genome that resides in the nuclei of infected hepatocytes. It serves as a key template for viral RNA transcription and is crucial for the persistence of HBV infection.

Although much progress has been made in HBV research, a significant limitation is the lack of suitable animal models that can accurately replicate the formation and maintenance of cccDNA. Mice, despite being one of the most commonly used laboratory animals due to their well-characterized immune system and ease of genetic manipulation, are not naturally susceptible to HBV infection. Consequently, alternative methods have been developed, including the hydrodynamic injection (HDI) of HBV genomes into mice via adeno-associated virus (AAV) vectors [11]. However, these models often fail to establish chronic HBV infection due to difficulties in achieving stable and sustained viral replication, limiting their utility for studying cccDNA dynamics and testing potential therapeutic interventions [12].

Previous studies have demonstrated that mice infected with recombinant AAV-HBV (rAAV-HBV) can exhibit persistent HBV infection, showing promise as a potential model for chronic hepatitis B (CHB) [13,14]. However, the rAAV-HBV mouse model uses genotype D, and there is a lack of mouse model utilizing genotype C HBV. Therefore, this study aimed to establish a mouse model that produces cccDNA using genotype C HBV.

Consequently, developing a robust mouse model that supports both the formation and maintenance of cccDNA is essential for advancing HBV research and evaluating novel therapeutic approaches aimed at curing chronic HBV infection.

Unlike other forms of HBV DNA, cccDNA is highly resistant to current antiviral therapies, making it a central challenge in efforts to cure HBV [15]. Even in patients who have achieved functional cure, cccDNA can remain detectable in liver tissue, contributing to the risk of viral reactivation [16]. This persistence highlights the critical need for therapeutic strategies that specifically target cccDNA, which plays a pivotal role in maintaining chronic HBV infection. We focused on detecting cccDNA through qRT-PCR by treating liver tissues with PASD (Plasmid-Safe ATP-Dependent DNase). Additionally, we aimed to confirm cccDNA through Southern blotting utilizing the Hirt method.

In this study, we aimed to establish a mouse model that not only demonstrates persistent HBV infection but also allows for the reliable detection and quantification of cccDNA in infected hepatocytes. By using an rAAV-HBV1.3 vector in C57BL/6 mice, we sought to create a model that accurately reflects the chronic phase of HBV infection and provides a valuable tool for studying the role of cccDNA in disease progression and treatment. We hypothesize that this model will demonstrate the successful establishment of such a model and its potential use in evaluating new therapeutic strategies targeting cccDNA.

## 2. Materials and Methods

### 2.1. rAAV Production

**Figure 1:**
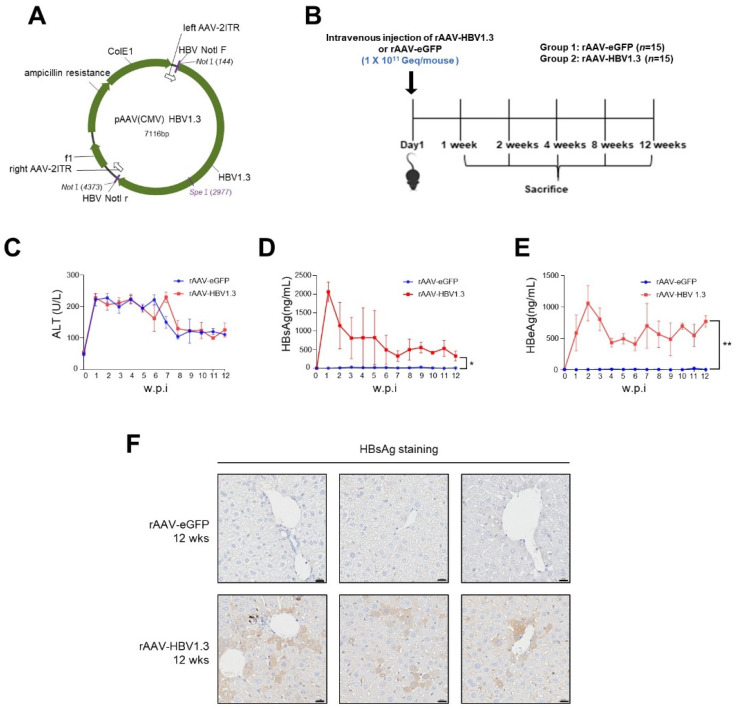
(**A**) pAAV-HBV1.3 plasmid map; (**B**) Group 1 was injected with rAAV-eGFP (1 × 10^11^ Geq/mouse) via the tail vein and served as the control group, while Group 2 was injected with rAAV-HBV1.3 (1 × 10^11^ Geq/mouse) via the tail vein. Three mice were euthanized at each of the following time points: week 1, week 2, week 4, week 8, and week 12; (**C**) ALT levels demonstrated by each week (**D**) HBsAg levels in serum by each week; (**E**) HBeAg levels in serum by each week; (**F**) Immunohistochemical staining of HBsAg. w.p.i.: weeks post-injection. Scale bars: 20 μm. AAV, Adeno-assoicated virus; eGFP, enhanced green fluorescent protein; HBsAg, HBV surface antigen; HBeAg, Hepatitis B e antigen; HBV, Hepatitis B virus; wks, weeks. * *p* < 0.05, ** *p* < 0.01.

Plasmid AAV-HBV 1.3 (pAAV-HBV1.3, 1.3-fold genome) containing a greater-than-genome-length HBV genotype C fragment derived from a Korean patient (nt 1073–3215–2067; GenBank: DQ683578.1) was constructed (Figure 1A). The rAAV8-HBV vectors carrying 1.3 copies of the HBV genome (genotype C) were produced from VectorBuilder (Republic of Korea) using pAAV-HBV1.3.

### 2.2. Animals

Male C57BL/6 mice (six-weeks-old) were purchased from Orient Bio Inc. (Seongnam, Republic of Korea). Each experimental group (rAAV-eGFP and rAAV-HBV1.3) consisted of 15 mice, with 3 mice per group sacrificed at weeks 1, 2, 4, 8, and 12. The rAAV-HBV1.3 mouse model was generated by administering vector genome equivalents of rAAV-HBV1.3 (1 × 10^11^ vg diluted in 200 µL PBS) via tail vein injection. The control group was administered intravenous injection of rAAV-eGFP (1 × 10^11^ vg diluted in 200 µL PBS). Mice were anesthetized via intraperitoneal injection with a mixture of Zoletil (50 mg/mL) and Rompun (23.32 mg/mL) at a dose of 0.1 mL/10 g body weight. Blood samples were collected weekly using the retro-orbital sinus method, and centrifuged at 13,000 rpm for 15 min at 4 °C to obtain serum. All animal care and experimental procedures followed the guidelines of the Institutional Animal Care and Use Committee and were approved by the Center for Medical Science of the Catholic University of Korea (2023-0153-01).

### 2.3. Serum Analyses

Serum HBsAg and HBeAg levels were analyzed using ELISA kits (Wantai, Biofarm). The serum was measured using a 100-fold dilution with PBS. Serum ALT levels were measured using a chemical analyzer (Catalyst One Chemistry Analyzer; IDEXX Laboratories, Westbrook, ME, USA) according to the manufacturer’s protocol.

### 2.4. Preparation of Liver Tissues

Liver tissues from sacrificed mice at weeks 1, 2, 4, 8, and 12 were cut and placed in cryovials, then stored in liquid nitrogen for future use. The mouse liver tissues were stored at approximately 25 mg each for qRT-PCR and approximately 100 mg each for Southern blot analysis.

### 2.5. Quantification of Total HBV DNA and cccDNA Copy Numbers by qRT-PCR

Total HBV DNA and cccDNA levels in liver tissues were measured by qRT-PCR, as previously described [17]. Total DNA was extracted from the resected liver tissue (approximately 25 mg) using the DNeasy Blood & Tissue Kit (Qiagen, Germantown, MD, USA), following the manufacturer’s instructions. In summary, 25 mg of liver tissue excised each week was added to ATL buffer (Qiagen, USA) and incubated overnight at 56 °C. Subsequently, the tissues are homogenized using disposable homogenizers. A TaqMan probe analysis was performed to detect HBV DNA expression. Total DNA was quantified at a concentration of 500 ng, and a total volume of 20 µL was used, which included the probe and master mix (Roche, Mannheim, Baden, Germany). Target DNA levels were determined by TaqMan gene expression assays (Applied Biosystems, Foster City, CA, USA). The assay IDs for each gene were Actb (Mm02519580_g1; Thermo Fisher Scientific, Waltham, MA, USA) and HBV DNA (Vi03453405_s1; Thermo Fisher Scientific). This process was performed using a Light Cycler 480 II (Roche Diagnostics). To detect cccDNA, total DNA was treated with PASD (LGC Biosearch Technologies, Hoddesdon, Hertfordshire, UK). DNA extraction was performed using 500 ng of total DNA with the following components: 10U PASD, 8 µL of 25 mM ATP, 20 µL of 10 × buffer, and ultra-pure water to a final volume of 50 µL. The mixture was incubated at 37 °C for 1 h, followed by heat inactivation at 70 °C for 30 min. cccDNA levels in the liver tissues were quantified using TaqMan gene expression analysis, with mitochondrial gene ND2 (Mm04225288_s1) serving as the internal control. cccDNA was detected using a forward primer (CCGTGTGCACTTCGCTTCA), reverse primer (GCACAGCTTGGAGGCTTGA), and probe ([6FAM] CATGGAGACCACCGTGAACGCCC [BBQ]). The following cycling conditions were applied: 1 cycle at 95 °C for 10 min, followed by 45 cycles of 95 °C for 10 s and 60 °C for 1 min. Quantification was performed using a serial dilution of a plasmid containing the HBV genome (nt 1073–3215–2067; GenBank: DQ683578.1) as the standard. The quantity of ccc DNA was calculated by interpolating the Ct value against the standard curve derived from standard DNA ranging from 0 to 10^7^ copies.

### 2.6. Immunohistochemistry

Liver tissues were fixed in 4% paraformaldehyde for one day and embedded in paraffin. After paraffin tissue sectioning, deparaffinization is performed. To prevent endogenous peroxide activity, sections were incubated in methanol containing 3% H_2_O_2_ for 30 min. Subsequently, the specimens were incubated in citrate buffer (0.01 M, pH 6.0) at 121 °C for 20 min using a microwave (RHS-1, Milestone, Grassobbio, Bergamo, Italy). The sections were stained with anti-HBsAg (Invitrogen, Waltham, MA, USA, Cat# MA5-13059) and incubated overnight at 4 °C. Subsequently, the slides were washed three times with PBS-T (Phosphate-Buffered Saline with Tween 20, pH 7.4) and incubated with EnVision Detection Systems Peroxidase/DAB, Rabbit/Mouse (#Cat K5007, Dako, Santa Clara, CA, USA) at 4 °C for 45 min. The DAB chromogen was added for 30 s, followed by counterstaining with hematoxylin.

### 2.7. Southern Blot

Hirt DNA extraction was performed according to previously described [17], with minor modifications. Briefly, resected liver tissues (100 mg) were lysed using Hirt lysis buffer containing 50 mM Tris-HCl (pH 7.5), 10 mM EDTA, 150 mM NaCl, and 1% SDS. Following high-salt precipitation with 2.5 M KCl and phenol-chloroform extraction, DNA was recovered by isopropanol precipitation. The cccDNA was further purified by ethanol precipitation and resuspended in Tris-EDTA (TE) buffer. HBV DNA detection was performed using a probe consisting of seven digoxigenin (DIG)-labeled fragments spanning the entire HBV genome [14]. The results were analyzed using Multi-Gauge Software v3.0 (Fujifilm, Tokyo, Japan)

### 2.8. Statistical Analysis

Statistical analyses were performed using the multiple *t*-test via the GraphPad Prism 8 software (GraphPad Software Inc., San Diego, CA, USA). The significance level was set at * *p* < 0.05, ** *p* < 0.01.

## 3. Results

### 3.1. rAAV-HBV1.3 Induces Long-Term HBV DNA Persistence in Mice

In this study, we developed a chronic HBV mouse model using rAAV-HBV1.3, which was administered via intravenous injection to C57BL/6 mice. The mice were observed over a 12-week period to monitor HBV infection and liver damage (Figure 1B). To assess liver injury, serum ALT levels were measured at different time points. Baseline ALT levels (week 0) were within normal range prior to rAAV administration. In both the rAAV-HBV1.3 and rAAV-eGFP groups, ALT levels were elevated until week 8, then returned to normal levels from week 9 (Figure 1C). The early increase in ALT levels is likely due to the acute inflammatory response induced by the intravenous injection rather than the viral infection itself. This transient rise in ALT levels subsided by week 9, indicating a resolution of the initial acute liver stress.

HBsAg and HBeAg levels in the serum were also measured. HBsAg levels peaked at week 1 and then gradually decreased but remained detectable until week 12 (Figure 1D), suggesting sustained viral antigen production. HBeAg levels peaked at week 2 and were similarly maintained at considerable levels until week 12 (Figure 1E), reinforcing the idea that viral replication persisted during this period. Immunohistochemistry confirmed the presence of HBsAg in hepatocytes up to week 12 (Figure 1F), supporting the chronicity of the infection. Neither HBsAg nor HBeAg was detectable at baseline (week 0).

### 3.2. Detection of HBV DNA and cccDNA in the rAAV-HBV1.3 Mouse Model

**Figure 2:**
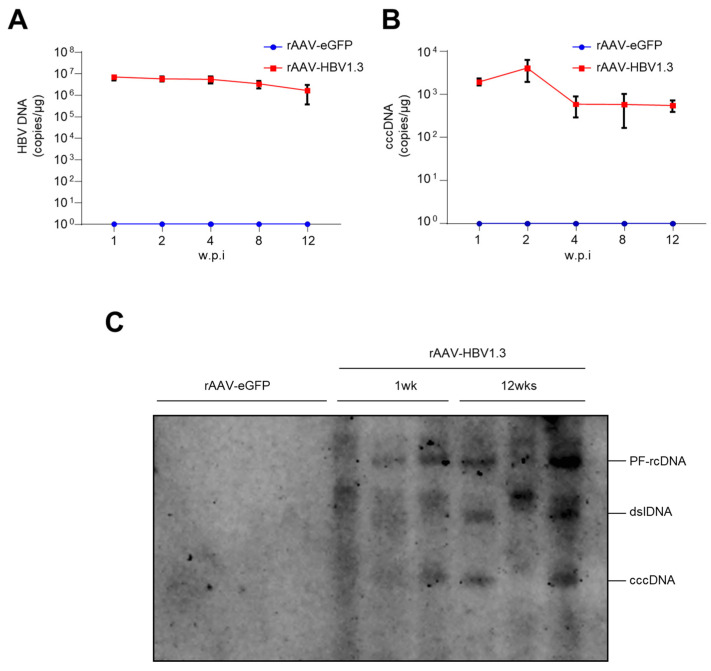
(**A**) HBV DNA copies/µg of liver tissues measured by qRT-PCR. (**B**) cccDNA copies/µg of liver tissues measured by qRT-PCR (**C**) cccDNA levels in mouse liver tissues at week 1 and week 12 measured by Southern blot. w.p.i, weeks post-injection; AAV, Adeno-assoicated virus; eGFP, enhanced green fluorescent protein; cccDNA, covalently closed circular DNA; HBV, Hepatitis B virus; PF-rcDNA, protein-free relaxed circilar DNA; dslDNA, double-strand linear DNA.

To further characterize HBV infection in the rAAV-HBV1.3 mouse model, we quantified intrahepatic HBV DNA levels over a 12-week period using qRT-PCR. As shown in Figure 2A, HBV DNA levels peaked at week 1 (approximately 6.9 × 10^6^ copies/µg of mouse liver tissues) and remained detectable until week 12, indicating persistent viral replication. Crucially, we detected cccDNA in liver tissues from rAAV-HBV1.3 mice. Figure 2B shows cccDNA levels peaked at week 2 (approximately 4070 copies/µg of mouse liver tissues) and maintained detectable levels (approximately 552 copies/µg of mouse liver tissues) throughout the 12-week period. This persistence of cccDNA mirrors the clinical scenario of chronic HBV infection. The detection of cccDNA is particularly important because it directly links our model to the pathophysiology of chronic HBV infection. The peak of cccDNA at week 2 likely reflects the early phase of HBV replication when cccDNA formation is most active. Its subsequent persistence indicates the stability of this episomal DNA, which remains a major therapeutic challenge in curing chronic HBV infection. Despite individual variation among mice, all rAAV-HBV1.3-injected mice showed HBV DNA and cccDNA expression. Southern blot analysis was performed to examine PF-rcDNA, dslDNA, and cccDNA levels in rAAV-HBV1.3 mice at weeks 1 and 12 (Figure 2C). We detected PF-rcDNA and dslDNA in liver tissues of the rAAV-HBV1.3 mouse model. Furthermore, cccDNA was present at week 1, with increased levels observed at week 12. The persistent presence of cccDNA suggests the successful establishment of an HBV infection mouse model.

## 4. Discussion

Chronic hepatitis B (CHB) remains a significant global health challenge due to the persistence of covalently closed circular DNA (cccDNA), a key viral reservoir that sustains infection despite antiviral therapy [18]. Achieving a complete cure, which requires the clearance of HBsAg and the elimination of all forms of HBV DNA, including cccDNA, is extremely difficult. Current treatment goals focus on functional cure, defined by the clearance of HBsAg and undetectable serum HBV DNA levels [19,20]. However, even in patients who achieve functional cure, inactive cccDNA or integrated HBV DNA can remain in hepatocytes, posing a risk of viral reactivation, especially in cases of immune suppression [21].

The persistence of cccDNA underscores its importance as a therapeutic target for CHB management. However, the development of robust animal models for cccDNA research has been hampered by the narrow species specificity of HBV, limiting the availability of suitable models. Although mice are widely used in research due to their convenience, they cannot be naturally infected with HBV because murine NTCP, the receptor critical for HBV entry, does not facilitate the internalization of the virus. [22].

This lack of a fully susceptible mouse model has been a major obstacle in studying the full HBV life cycle, particularly the formation and persistence of cccDNA.

In a study by Zaichao et al., cccDNA formed during the transduction of AAV-HBV1.04 was not derived from rcDNA but shared the same sequence as that observed in the sequencing data of wild-type HBV cccDNA [13]. Additionally, in rAAV-HBV1.2 and rAAV-HBV1.3 mouse models, viral proteins can be generated from both the viral episome and cccDNA [14,23]. These models have proven valuable for studying chronic HBV infection and cccDNA dynamics.

Our study builds on this approach by successfully establishing a chronic mouse model of HBV infection using a recombinant AAV carrying the HBV genome, rAAV-HBV1.3. This model supports sustained HBV replication and cccDNA formation, mimicking key aspects of chronic infection in humans. We observed that HBV DNA levels peaked early, at week 1, and remained stable for 12 weeks, indicating continuous viral replication. Crucially, cccDNA levels peaked at week 2 and persisted throughout the study period, confirming the long-term presence of this viral reservoir. The persistence of cccDNA in our model is particularly significant, as it mirrors the clinical scenario where cccDNA remains detectable in patients even after serum HBV DNA becomes undetectable. This makes our model particularly relevant for studying therapeutic strategies targeting cccDNA.

The genotype-specific nature of HBV also plays a crucial role in disease progression and response to therapy. Genotype C, the most prevalent genotype in South Korea [24], is associated with more severe disease progression compared to other genotypes such as B. Our model, using rAAV-HBV1.3 with genotype C, thus provides a valuable platform for studying the pathophysiology of HBV in the Korean context. Previous studies have demonstrated cccDNA expression in mice infected with rAAV-HBV1.3 using Southern blot analysis, which aligns with our findings showing detectable cccDNA in liver tissues of infected mice [23].

Our results are distinct from previous HDI-based HBV models that injected plasmid, where cccDNA was not reliably detected, and HBsAg was maintained at lower levels [25]. In contrast, our rAAV-HBV1.3 mouse model demonstrated sustained high levels of HBsAg in serum and detectable cccDNA throughout the 12-week period. This suggests that our model more closely replicates the chronic nature of HBV infection, making it a valuable tool for long-term studies of CHB pathogenesis and for evaluating new antiviral therapies aimed at eradicating cccDNA.

While qRT-PCR signals from liver tissue could not definitively distinguish between HBV DNA and cccDNA, the absence of signals in the negative control group supports the validity of our results. In Southern blot analysis, some liver tissue samples from rAAV-HBV-infected mice at week 12 showed no detectable cccDNA, suggesting an absence of cccDNA formation in the hepatocytes of these excised tissue samples. Additionally, our model demonstrated normal serum ALT levels by week 12, suggesting minimal liver damage at later stages of infection, despite the persistence of viral markers such as HBsAg and HBeAg. Both HBsAg and HBeAg are associated with cccDNA expression and can serve as surrogate markers for ongoing viral activity [26].

Additionally, there is a discrepancy in the detection of cccDNA in liver tissue samples from rAAV-HBV-infected mice at week 12 between qRT-PCR and Southern blot analyses. Different tissues used for qRT-PCR and Southern blot might have different levels of cccDNA.

rAAV-HBV1.3 supports persistent HBV infection and cccDNA expression over an extended period. This model provides a valuable platform for studying the dynamics of cccDNA in chronic HBV infection and for evaluating novel therapeutic strategies aimed at eradicating this critical viral reservoir. Future studies should focus on refining detection techniques and exploring therapeutic interventions targeting cccDNA to move closer to a complete cure for CHB.

## Data Availability

The datasets generated for this study are available on request to the corresponding author.
